# A Unique Hospital Chapel

**Published:** 1914-04-25

**Authors:** 


*To the Editor of* The Hospital.

### A Unique Hospital Chapel.

*To the Editor of* The Hospital.

Sir,—The very interesting article by the Rev. B. Rees in your issue of April 18, in which particulars are given of several of the principal institutional chapels of London, prompts me to mention one which, though little known, is in some ways the most notable of them all. St. Saviour's Hospital, Osnaburgh Street, contains a small chapel for the use of the Sisterhood by whom the institution is managed and of the patients in the hospital. The whole of this chapel is filled with really magnificent mediæval carved woodwork, which was imported, I believe, into this country some half-century or more ago from Belgium, its original home. It is some years since I last had the opportunity of seeing this beautiful work of art; but, speaking from memory, I fancy I am right in saying that it was done by monks in the late sixteenth or early seventeenth century. It was in this chapel that the late Rev. A. Brinckman laboured. Although the chapel is comparatively little known, it is on its merits one of the "show-places" of London, and is certainly unique amongst institutional possessions. It is a great joy and pride to the Sisterhood, by whom it is regarded with affection and devotion.

Incidentally, I should like to testify to the valuable work which is done in St. Saviour's, one of the few private hospitals which provide accommodation for ladies whose means do not permit them to go to the ordinary nursing homes and yet are not suitable subjects for admission to free hospitals. I trust I am not indiscreet in recommending this valuable foundation to the generosity of philanthropic persons in search of suitable outlets for their liberality.—Yours faithfully, Physician.

